# Clinical application of computed tomographic volumetric imaging in postoperative lung function assessment in patients with lung cancer

**DOI:** 10.1186/s12880-024-01268-7

**Published:** 2024-04-29

**Authors:** Zhifu Xu, Xili Wang, Zhanxian Shen, Biao Shi, Yanni Zhang

**Affiliations:** 1 Department of CT Lab, ZhangJiaKou First Hospital, 075000 Zhangjiakou, Hebei China; 2 Department of Ultrasound, ZhangJiaKou First Hospital, 075000 Zhangjiakou, Hebei China; 3 Department of Respiratory Medicine, Gu Yuan Xian People’s Hospital, 075000 Zhangjiakou, Hebei China; 4 Department of Oncology, ZhangJiaKou First Hospital, No.6, Libaisi Lane, Qiaoxi District, 075000 Zhangjiakou, Hebei China

**Keywords:** Computed tomography, Volumetric imaging, Respiratory lung density, Postoperative lung function, Lung cancer, Complication occurrence

## Abstract

**Background:**

To evaluate the effectiveness of the computed tomographic (CT) volumetric analysis in postoperative lung function assessment and the predicting value for postoperative complications in patients who had segmentectomy for lung cancer.

**Methods:**

CT scanning and pulmonary function examination were performed for 100 patients with lung cancer. CT volumetric analyses were performed by specific software, for the volume of the inspiratory phase (Vin), the mean inspiratory lung density (MLDin), the volume of expiratory phase (Vex), and the mean lung density at expiratory phase (MLDex). Pulmonary function examination results and CT volumetric analysis results were used to predict postoperative lung function. The concordance and correlations of these values were assessed by Bland-Altman analysis and Pearson correlation analysis, respectively. Multivariate binomial logistic regression analysis was executed to assess the associations of CT data with complication occurrence.

**Results:**

Correlations between CT scanning data and pulmonary function examination results were significant in both pre- and post-operation (0.8083 ≤ *r* ≤ 0.9390). Forced vital capacity (FVC), forced expiratory volume in the first second (FEV1), and the ratio of FVC and FEV1 estimated by CT volumetric analyses showed high concordance with those detected by pulmonary function examination. Preoperative (Vin-Vex) and (MLDex- MLDin) values were identified as predictors for post-surgery complications, with hazard ratios of 5.378 and 6.524, respectively.

**Conclusions:**

CT volumetric imaging analysis has the potential to determine the pre- and post-operative lung function, as well as to predict post-surgery complication occurrence in lung cancer patients with pulmonary lobectomy.

## Background

Lung cancer remains one of the most lethal cancers worldwide, and also a most diagnosed cancer both in men and women [1]. Lung cancer accounts for 12% of all lung cancer cases in men and 13% of female cancers [2]. The greatest number of estimated cancer-related deaths is from lung cancer both in men and in women in the USA in 2023 [2]. From 1973 to 2016, China witnessed a gradual increase in the mortality rate of lung cancer [3]. Lung cancer has become the leading cause of cancer-related death for the last twenty years in China. Surgery is currently one of the most effective treatments for lung cancer [4]. Reasonable assessments of preoperative surgical risks and postoperative functional recovery is of great significance for patients’ post-surgery quality of life. For lung cancer patients undergoing pulmonary lobectomy, the important physiological function change after surgery is the decline of respiratory function. At present, the clinical routine pulmonary function test (PFT) is a standard examination method for measuring lung volume and evaluating lung function [5]. However, PFT itself has some shortcomings. For example, the measurement result of PFT is the combination of bilateral lung respiratory, instead of respective evaluations at the affected lung or lobe [6]. For some patients with imbalanced bilateral lung functionit may have a significant impact on the patient’s respiratory function and will inevitably affect the postoperative rehabilitation and quality of life, if the lobar with higher respiratory quality is removed due to insufficient preoperative evaluation.

Benefiting from the development of artificial intelligence, computer-aided detection tools have been commercially available for thoracic imaging, with fewer false-positive results per examination [7]. Computer-aided detection tools, especially computed tomography (CT), can obtain accurate tumor localization and electron densities. CT can assist in volumetric measurements of tumors and highlight signs of diseases within the lungs. A previous study revealed that characterization and stratification in lung cancer using low-dose CT has been shown to reduce lung cancer-specific mortality [8]. Moreover, volumetric analyses of CT-screen may provide useful information in the follow-up and management of CT-detected lung cancers [9].

To assess the effect of CT volumetric imaging in lung cancer patients who receive surgery, PFT and commercially available CT were simultaneously used for detecting lung function pre-operation and post-operation, to investigate the performance of CT volumetric analyses in lung function.

## Methods

### Patients

Patients who were diagnosed as primary lung cancer and hospitalized in the thoracic surgery department of our hospital for lobectomy were enrolled from January 2022 to November 2022. The selection criteria are as follows:

1. Patients with lung cancer diagnosed pathologically by biopsy or fiberoptic bronchoscopy before operation and without distant metastasis;
2. Patients who are able to bear the surgery after preoperative examination and evaluation and have no contraindications to traditional lung function tests such as severe pulmonary bullae or infectious diseases;
3. Patients who have no obvious hearing impairment and can cooperate with breathing training to complete inspiratory and expiratory examinations under CT examination.

Exclusion criteria included:

1. Patients with prior chemotherapy, major surgery history, or any radiation therapy within the last 21 days;
2. Patients with any malignancy within the last 5 years; patients with any severe and unstable medical comorbidities;
3. Patients with a history of occupational dust exposure and other diseases that affect lung function.

Patients were enrolled with written, informed consent. The study was approved by the institutional review board of ZhangJiaKou First Hospital (no. 2,021,039).

### CT examinations

Scans in this study were acquired on the GE CT Revolution CT (General Electric, United States). CT scans of the chest were performed one week before surgery and three months after surgery. The patients underwent respiratory training before scanning. The patient was placed in a supine position, with both arms raised above the head, and then held their breath at the end of deep inhalation or the end of deep exhalation. Scanning was performed from the apex to the bottom of the lungs at the end of deep inhalation and the end of deep exhalation, respectively. Tube voltage was 120 kV, tube current was set to automatic mode (Smart mA: 50-650 mA), the pitch was 0.984:1, and rotation time was 0.6 s. The scanning layer thickness was set to 5 mm. In chest CT images, we reconstructed a moderately smooth thin layer lung window image and transferred it into the 3D card. Then we set the range of regional growth to -1000 for the trachea, which was the air. And then we placed the seeds in the center of the trachea, add items, and the trachea tree was displayed.

### Imaging segmentation and quantitative measurements

The images were sent to the GE Advantage Workstation (AW4.7; GE Healthcare), and Thoracic VCAR, a lung function analysis software, was used to quantitatively analyze images. We used automatic segmentation and threshold limiting (upper limit: -200HU, lower limit: -1024HU) techniques to segment lung tissue from other soft tissues, to obtain a three-dimensional model of lung tissue [10, 11]. Then, the interlobular fissures were identified to divide the five lobes (left upper lobe (LUL), left lower lobe (LLL), right upper lobe (RUL), right middle lobe (RML), and right lower lobe [RLL]) by automatic segmentation, assisted by manual corrections when segmentation was inaccurate. From the images of the inspiratory phase, the volume of inspiratory phase (Vin) and the mean inspiratory lung density (MLDin) were obtained. From the images of the expiratory phase, the volume of expiratory phase (Vex) and mean lung density at expiratory phase (MLDex) were obtained. The lung volume change was defined as the difference between Vin and Vex. Each case was measured three times, and the final value included in the results was the average of the three measurements.

### Pulmonary function measurement

The lung function examination was completed on the same day as CT examination. PFT was measured at a MasterScreen pulmonary function meter (Vyaire Medica, Hoechberg, Germany). The measured indicators included the total lung capacity (TLC), residual volume (RV), forced vital capacity (FVC), forced expiratory volume in the first second (FEV1), and the ratio of FVC and FEV1 (FEV1/FVC) of patients before and after surgery.

### Prediction of lung function

After manually specifying the portion of the lung to be removed, the volume of the designated segment and the total lung volume of the individual patient were calculated on the GE Advantage Workstation (AW4.7; GE Healthcare). Then, previous formulae were used to calculate the predicted values of FVC and FEV1 [12]:

PredictedFVC = preFVC×[(total lung volume-resected lung volume)/total lung volume]

PredictedFEV1 = preFEV1×[(total lung volume-resected lung volume)/total lung volume]

### Statistical analysis

The data were statistically analyzed using SPSS 20.0 and GraphPad Prism software, presented as mean ± standard deviation. The correlation analyses between the indicators obtained from the CT volumetric test and the measured lung function were performed using Pearson correlation analysis. The limits of agreement for predicted parameters and scanned values were determined using Bland-Altman analysis. Multivariate binomial logistic regression analysis was executed to assess the associations of potential variables with complication occurrence. The difference between actual postoperative parameter values and values estimated by CT volumetric analysis in different lung lobes before surgery was conducted using one-way ANOVA followed by Dunnett-t test, and the pairwise comparison was conducted using the Student-Newman-Keuls method. *P* < 0.05 indicated a statistically significant difference.

## Results

### Association between ventilation indexes and parameters obtained by CT volumetric analysis before surgery

**Fig. 1:**
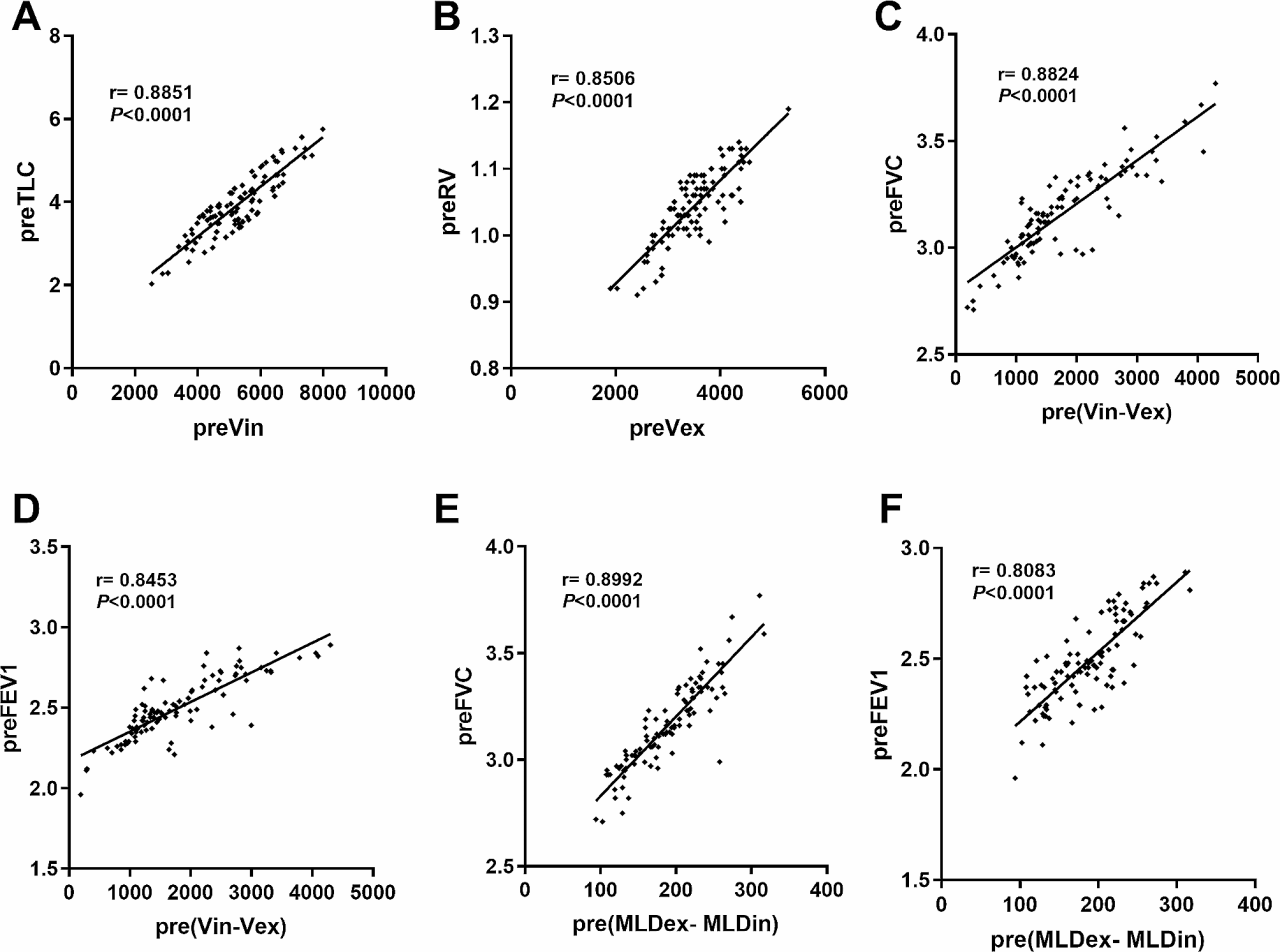
The correlations of the preoperative measurements results between traditional pulmonary function examination and Revolution CT volumetric imaging analysis, including preoperative total lung capacity (preTLC) versus preoperative inspiratory total lung volume (preVin) (**A**), preoperative residual volume (preRV) versus preoperative expiratory total lung volume (preVex) (**B**), preoperative lung volume change [pre(Vin-Vex)] versus preoperative forced vital capacity (preFVC) (**C**), pre(Vin-Vex) versus preoperative forced expiratory volume in the first second (preFEV1) (**D**), preoperative mean lung density change [pre(MLDex- MLDin)] versus preFVC (E), and pre(MLDex- MLDin)] versus preFEV1.

One hundred individuals were enrolled in this study and the clinical characteristics were summarized in Table 1. Based on the Pearson correlation coefficients, a positive correlation was observed between the preoperative inspiratory total lung volume (preVin) and preoperative total lung capacity (preTLC) (*r* = 0.8851, *P* < 0.001; Fig. 1A), as well as between the preoperative expiratory total lung volume (preVex) and residual volume (preRV) (*r* = 0.8506, *P* < 0.001; Fig. 1B). In addition, the preoperative total lung volume change, pre(Vin-Vex), was significantly related to the preoperative FVC (*r* = 0.8824, *P* < 0.001; Fig. 1C) and preoperative FEV1 (*r* = 0.8453, *P* < 0.001; Fig. 1D). Furthermore, preoperative mean lung density change from the inspiratory phase to the expiratory phase (MLDex-MLDin) was correlated with preoperative FVC (*r* = 0.8992, *P* < 0.001; Fig. 1E) and FEV1 (*r* = 0.8083, *P* < 0.001; Fig. 1F).

**Table 1:** Patient characteristics

Clinicopathological parameters	Cases number (n)
**Total**	100
**Age (years)**	
≦ 60	46
>60	54
**Gender**	
Female	39
Male	61
**Smoking history**	
never	51
Ex-smoker	40
Current smoker	9
**Clinical stage**	
I	82
II	15
III	3
**Number of resected segments**	
1	34
2	43
3	16
4	7
**Location of resected segment**	
Right upper lobe	18
right lower lobe	47
left upper lobe	21
left lower lobe	14

### Association between ventilation indexes obtained by CT volumetric analysis after surgery

**Fig. 2:**
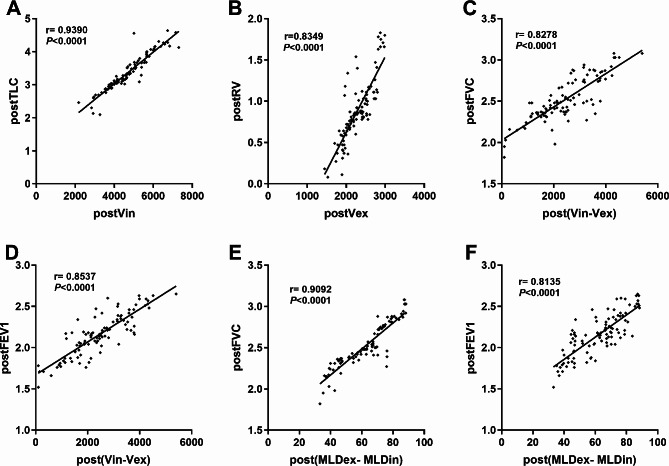
The correlations of the postoperative measurements results between traditional pulmonary function examination and Revolution CT volumetric imaging analysis, including postoperative total lung capacity (postTLC) versus postoperative inspiratory total lung volume (postVin) (A), postoperative residual volume (postRV) versus postoperative expiratory total lung volume (postVex) (B), postoperative lung volume change [post(Vin-Vex)] versus postoperative forced vital capacity (postFVC) (C), post(Vin-Vex) versus postoperative forced expiratory volume in the first second (postFEV1) (D), postoperative mean lung density change [post(MLDex- MLDin)] versus postFVC (E), and post(MLDex- MLDin)] versus postFEV1.

After surgery, we analyzed the association between ventilation parameters of the 100 patients obtained by CT volumetric analysis. There was a good correlation between TLC and Vin (Pearson coefficient of 0.9390, *P* < 0.0001; Fig. 2A), as well as between Vex and RV (Pearson coefficient of 0.8349, *P* < 0.0001; Fig. 2B). Pearson correlation analysis between (Vin-Vex) and FVC revealed a positive Pearson r of 0.8278 (*P* < 0.0001; Fig. 2C). Pearson correlation analysis between (Vin-Vex) and FEV1 revealed a positive Pearson r of 0.8537 (*P* < 0.0001; Fig. 2D). Additionally, the Pearson r between (MLDex-MLDin) and FVC was 0.9092 (*P* < 0.0001; Fig. 2E), and the Pearson r between (MLDex-MLDin) and FEV1 was 0.8135 (*P* < 0.0001; Fig. 2F).

### Concordance of predicted and measured postoperative pulmonary function by computed tomographic volumetric analysis

**Fig. 3:**
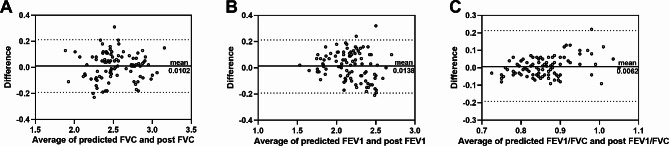
Bland-Altman plots comparing the predicted forced vital capacity (FVC) (A), forced expiratory volume in the first second (FEV1) (B), and the ratio of FVC and FEV1 (FEV1/FVC) (C) with actually measured lung function by traditional pulmonary function examination

The limits of concordance for each actual and predicted postoperative FVC, FEV1, and FEV1/FVC were assessed by Bland-Altman analysis. The results revealed that the mean bias for predicted and measured postoperative FVC was 0.0102 (95% limits of agreement: -0.1922 to 0.2126; Fig. 3A). Moreover, the predicted FEV1 values from the prediction were the closest to the observed values (Bias: -0.0138, 95% limits of agreement: -0.2260 to 0.1984; Fig. 3B). The 95% limit of agreement for predicted and actual postoperative FEV1/FVC was from − 0.1156 to 0.1032 (Bias: -0.0062, Fig. 3C).

### Predicting significance of preoperative (Vin-Vex) and (MLDex- MLDin) for complication occurrence

**Fig. 4:**
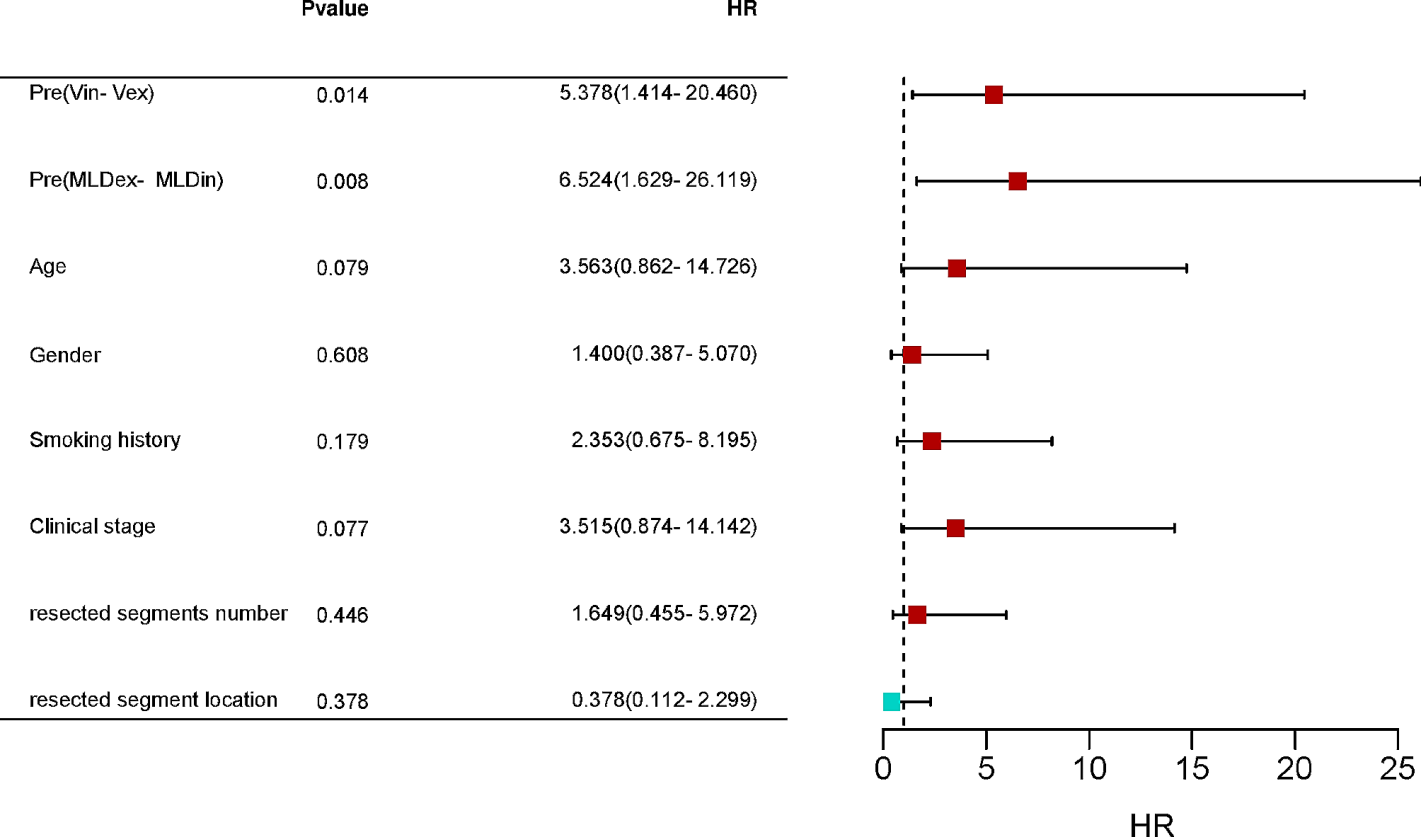
Forest plot for hazard ratio of Multivariate binomial logistic regression analysis

Multivariate logistic regression analysis was used to screen the risk factors for complication occurrence after surgery. The results (Table 2; Fig. 4) identified preoperative (Vin-Vex) and (MLDex- MLDin) values as significant predictors, with hazard ratios of 5.378 [95% Confidence Interval (CI): 1.414–20.460, *P* = 0.014] and 6.524 (95% CI: 1.629–26.119, *P* = 0.008), respectively.

**Table 2:** Multivariate Binomial Logistic Regression Analysis of significant risk factors for postoperative complications

Parameters	HR	95%CI	P
Preoperative Vin-Vex	5.378	1.414–20.460	0.014
Preoperative MLDex- MLDin	6.524	1.629–26.119	0.008
Age	3.563	0.862–14.726	0.079
Gender	1.400	0.387–5.070	0.608
Smoking history	2.353	0.675–8.195	0.179
Clinical stage	3.515	0.874–14.142	0.077
resected segments number	1.649	0.455–5.972	0.446
resected segment location	0.378	0.112–2.299	0.378

## Discussion

Radiology plays an extremely important role in the management of cancer patients, as it can accurately and describe the condition and organ status of cancer in detail thus assisting in treatment plan making [13]. At present, CT examination has become a common imaging examination method and is gradually accepted by a large number of patients [14]. The lung function assessment technology derived from CT technology will undoubtedly have its unique technical advantages and operability [15]. The traditional pulmonary function examination is an important method to evaluate the preoperative pulmonary function of patients undergoing lobectomy or pneumonectomy in thoracic surgery. Combined with the results of arterial blood gas analysis, it can determine whether the patient can tolerate the operation and greatly reduce the postoperative risk [16]. However, given the complex of traditional pulmonary function examination, simple and reliable examinations, such as CT, are undoubtedly of great significance for the assessment of surgical risks or prediction for post-surgery lung function.

CT volumetric analyses have been emerging as an important tool clinically [17]. This study assessed the clinical application of CT volumetric imaging in postoperative pulmonary ventilatory assessment in patients with lung cancer. Our results confirmed the reliability of CT volumetric analyses in pulmonary ventilatory assessment, with multiple indexes showing correlation coefficient more than 0.8 between the CT data and pulmonary ventilation data. In addition, CT parameters, Vin and Vex, were related to the TLC and RV, respectively, both pre- and post-operation. The results of (Vin-Vex) showed a positive relation to FVC and FEV1 obtained from the traditional pulmonary function examination in our patients. Terefore, there was a high agreement of the results from CT volumetric analyses with those from traditional pulmonary function examination.

Then, this present study explored the predictability of predicted postoperative pulmonary function (FVC and FEV1) estimated by traditional pulmonary function examination and CT volumetric imaging analysis in patients who underwent segmentectomy. A previous study has reported that the agreement between predicted postoperative pulmonary function estimated by anatomical segment counting and CT volumetric analyses was quite high for early-stage lung cancer [12]. Our results demonstrated that there were good concordances between actual postoperative FVC and FEV1 values and the values calculated by CT volumetric analysis. Notably, the lobar volume change was decreased in almost all lobes, which has been reported by Bae et al. [12]. In addition, Logistic regression analysis demonstrated that the preoperative lung volume change (Vin-Vex) and mean lung density change (MLDex- MLDin) were independent predictors of postoperative complications. This was in line with the results of a previous study, which demonstrated the inspiratory/expiratory CT volumetry data can predict postoperative pulmonary function after lobectomy for lung cancer [18]. A previous comparative study also supports the reliability and accuracy of volumetric computed tomography in predicting postoperative lung function, compared with segment counting or scintigraphy [19]. Since our study’s sample size is relatively limited and limited to one single institution, more research in multi-institutions with larger datasets will be required to confirm our findings.

## Conclusions

In conclusion, the lung function detected from CT volumetric analysis are consistent with those from the lung function measurements in lung cancer patients. In addition, CT volumetric imaging can be a future tool to predict post-operative lung function and to predict the postoperative complications of patients. This study may propose important implications for improving the prediction of patients’ post-operative lung function and ultimately contributing to better patient outcomes.

## Data Availability

No datasets were generated or analysed during the current study.

## Abbreviations

CT: Computed tomographic
Vin: Volume of inspiratory phase
MLDin: Mean inspiratory lung density
Vex: Volume of expiratory phase
MLDex: Mean lung density at expiratory phase
FVC: Forced vital capacity
FEV1: Forced expiratory volume in the first second
PFT: Pulmonary function test
LUL: Left upper lobe
LLL: Left lower lobe
RUL: Right upper lobe
RML: Right middle lobe
RLL: Right lower lobe
TLC: Total lung capacity
RV: Residual volume
